# Tongue metastasis as an initial presentation of renal cell carcinoma: a case report and literature review

**DOI:** 10.1186/1752-1947-2-249

**Published:** 2008-07-25

**Authors:** Faisal Azam, Muneer Abubakerr, Simon Gollins

**Affiliations:** 1 Department of Oncology, North Wales Cancer Treatment Centre, Glan Clwyd Hospital, Bodelwyddan, Rhyl, LL18 5UJ, UK

## Abstract

**Introduction:**

Primary tumour of the kidney metastasizing to the tongue is very unusual and only anecdotal cases have been reported. An exhaustive literature review covering the period from 1911 onwards disclosed 28 cases. Out of those, only 3 cases presented initially with tongue metastases before the diagnosis of primary renal cell carcinoma.

The prognosis for patients with lingual metastasis of renal cell carcinoma is poor. Treatment of tongue metastasis is usually palliative and aims to provide patient comfort by means of pain relief and prevention of bleeding and infection. Surgical excision is recommended as the primary treatment with emphasis on preservation of tongue structure and function.

**Case presentation:**

We report a case of tongue metastasis as an initial presentation of renal cell carcinoma in a 78-year-old man. Initially thought to be primary tongue cancer but on review of his histopathology again, it was diagnosed to be a rare metastasis from kidney cancer.

**Conclusion:**

Tongue metastasis from renal cell carcinoma is rare and its diagnosis is a challenge. The prognosis of patients with tongue metastasis is poor. Similar to the primary tumours of the tongue, metastatic lesions may be ulcerated or polypoid. Since the tongue is a rare metastatic site, when a lesion is detected, a thorough evaluation to distinguish between metastasis and primary cancer should be made as the management and prognosis vary.

## Introduction

Metastasis to the tongue seldom occurs, and lingual metastasis as an initial sign of cancer occurs even less frequently.

Metastasis to the head and neck area from a primary site in the abdomen is rare. Renal cell carcinoma (RCC) is the third most common tumour after lung and breast to metastasize to the head and neck region. Less than 15% of patients with renal cell carcinoma actually show metastasis to this area. We discuss a case of renal cell carcinoma presenting with pathologically proven metastasis in the tongue.

## Case presentation

A 78-year-old man who was a chronic smoker presented to the maxillofacial department at a district general hospital with a 6-week history of difficulty in swallowing solids together with pain in his pharynx.

On examination, he was noted to have a 3 × 2 cm solitary pedunculated lesion on the right side of the anterior two-thirds of his tongue crossing the midline. His tongue mobility was normal and there was no palpable cervical lymphadenopathy.

**Figure 1:**
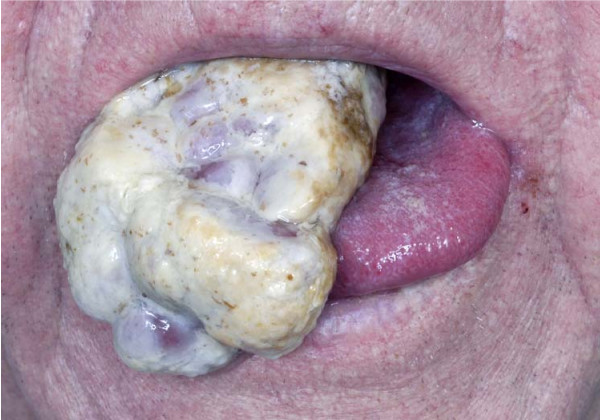
Tongue metastasis.

Systematic examination of chest, abdomen and heart were normal. The lesion was biopsied and initially reported as a primary squamous cell carcinoma with some clear cell changes. His blood tests including renal functions were normal. His case was discussed in the head and neck cancer multidisciplinary team (MDT) meeting and subtotal glossectomy was planned after a staging MRI (magnetic resonant imaging) scan followed by adjuvant radiotherapy to the head and neck region. While awaiting an MRI, he presented to the hospital with severe pain in his oral cavity and difficulty in swallowing. His tongue lesion had doubled in size in a matter of two weeks and was now protruding outside the mouth (Figure 1). It was considered unusual for primary squamous cell carcinoma of tongue to behave like that. The pathology was therefore reviewed at the same MDT meeting and this time the lesion was reported as partly squamous epithelium covered by fibromuscular tissue showing infiltration by a carcinoma, seen in the nests with extensive clear cell changes. The differential diagnosis was considered to be squamous cell carcinoma with clear cell changes, metastatic salivary gland neoplasm or metastases from RCC. It was decided to arrange an urgent CT scan and to debulk the tongue lesion surgically, to improve his symptoms. The patient had not described any suspicious urinary symptoms.

**Figure 2:**
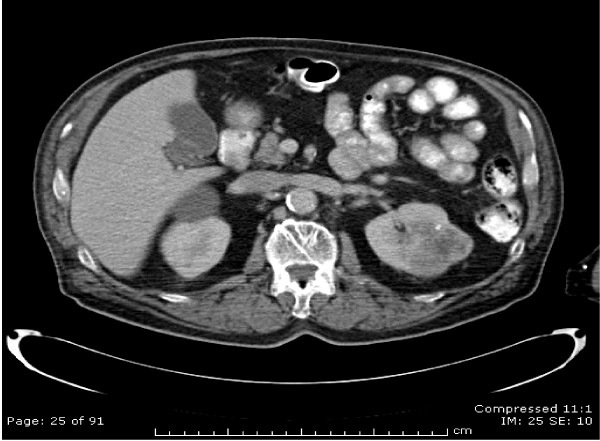
CT scan showing primary tumour of left kidney.

A CT scan of the neck, chest and abdomen revealed a 4.7-cm sized irregular mass in the left kidney suggestive of RCC (Figure 2). There was no local extension and the left renal vein was clear. A solitary tongue lesion with no neck nodes was reported. No metastases were seen in the lungs, liver, adrenals, spleen and bones.

As per the MDT decision, he underwent a debulking surgery of the tongue metastasis, which was performed without complication. His swallowing improved significantly. Postoperatively, he received radiotherapy to his oral cavity delivering a dose of 60 Grays in 30 daily fractions over 6 weeks, which was well tolerated. Radiotherapy was given to treat the microscopic disease in his head and neck region.

A post-radiotherapy CT scan, 18 weeks after initial presentation, was arranged before radical nephrectomy, which unfortunately revealed early evidence of lung metastases. As the patient reported shoulder pain, a plain X-ray and bone scan were carried out and this revealed evidence of a solitary bone metastasis in the right scapula.

Following his debulking surgery and adjuvant radiotherapy, he underwent a radical left-sided nephrectomy. Histopathology confirmed a Fuhrman grade 3 clear cell carcinoma of the left kidney with extension into the superior perirenal fat but not into the renal sinus and with no microvascular infiltration. The maximal dimension of the tumour was 5 cm. The patient has subsequently been treated with interferon-alpha (dose: 3 MU, three times a week) as a systemic treatment for his metastatic disease. A repeat CT scan after six months of treatment showed a complete response with no evidence of any distant metastases.

## Discussion

RCC may remain clinically occult for most of its course. The classic presentation of pain, haematuria, and flank mass occurs in a minority of patients and is often indicative of advanced disease. A tumour in the kidney can progress unnoticed to a large size in the retroperitoneum until metastatic disease appears. It can metastasize to any location in the body, and its propensity to metastasize to unusual sites has been well documented. Approximately 30% of patients with renal carcinoma present with metastatic disease, 25% with locally advanced renal carcinoma, and 45% with localized disease [1]. About 75% of patients with metastatic renal carcinoma have metastases to the lung, 36% to soft tissues, 20% to bone, 18% to liver, 8% to cutaneous sites and 8% to the central nervous system [2]. Approximately 15% of renal cell carcinomas metastasize to the head and neck region – specifically, to the paranasal sinuses, larynx, jaws, temporal bones, thyroid gland, and parotid glands [3,4]. Tongue metastasis is rare.

After an exhaustive literature search, we found 28 cases which had been reported since 1911 (Table 1). Tongue metastasis as an initial presentation of RCC is extremely rare and we found only three cases published in the literature so far, reported in 1987, 1994 and 1996 (Table 2).

**Table 1:** Previous case reports of renal cell carcinoma metastasizing to tongue

S No	Author.	Year	Age/sex	Site	Other metastases
1	Kostenko	1911	43/M		
2	Coenen	1914	62/F		
3	McNattin & Dean	1931	58/M		Lung, heart, skin
4	Trinca & Willis	1936	57/M		
5	Schrag	1945	34/M		Lung
6	Del Carmen	1970	77/M		None
7	Satomi *et al.*	1974	41/F	Left surface	Lung
8	Friedlander	1979	84/M	Tip of tongue	Lung
9	Fitzgerald	1982	63/M	Right dorsum	Brain
10	Kitao *et al.*	1986	37/M	BOT	None
11	Inai	1987	42/M	Left base	Lung
12	Matsumota & Lio	1987	77/F	Left surface	Lung
13	Kapoor	1987	70/M	Not mentioned	Not mentioned
14	Madsion & Fereson	1988	63/M	Right ventral surface	Lung, liver
15	Ishikawa	1991	59/F		Lung, bone
16	Okabe *et al.*	1992	58/M	Left base	Lung, brain
15	Shibyama	1993	41/M	BOT	Lung, bone, lymph nodes
16	Ziyada	1994	59/M	Right base	None
17	Aguirre	1996	82/F	Tip of tongue	Brain
18	Airoldi	1995	51/M		Lung
19	Konya	1997	59/M		Para-aortic, lymph nodes
20	Tomita	1998	50/M	Left border	Lung, brain, skin
21	Goel	1999	62/M	Left surface	Lung
22	Navarro *et al.*	2000	62/M	Right lateral	Lung
23	Fukuda	2002	74/M	Left side	
24	Mariomi *et al.*	2002	87/F	Dorsum	Lung, liver, thyroid, pancreas
25	Emer *et al.*	2003	45/M	Tip of tongue	Nose, lungs
26	Kyan & Kato	2004	66/M	Base of tongue	Lungs
27	Torres-Carranza	2006	49/F	Middle third of dorsum	Lungs
28	Huang & Chang	2006	76/F	BOT	Lungs, liver
29	Present case	2007	68/M	Anterior right lateral	Lungs, bone

BOT, base of tongue.

**Table 2:** Tongue metastasis as the initial presentation of renal cell carcinoma

S No	Authors	Year	Age/sex	Site	Other metastases
1	Kapoor *et al. *[10]	1987	70/M	Not mentioned	Not mentioned
2	Ziyada *et al. *[11]	1994	59/M	Right BOT	None
3	Aguirre and Rinaggio [12]	1996	82/F	Tip of tongue	Brain
5	Present case	2007	68/M	Anterior right lateral	Lungs, bone

BOT, base of tongue.

Possible routes of metastatic spread to the tongue are the arterial, venous and lymphatic circulation. Metastases are mostly located on the base of the tongue possibly due to its rich vascular supply, through the dorsal lingual artery, and due to immobility as compared to other parts of the tongue. RCC invades the local vascular network of the kidney and spreads through the systemic circulation. Head and neck metastasis is commonly associated with lung metastases. If there are no signs of pulmonary disease, as in our case initially, it is possible that spread has been via Batson's venous plexus or via the thoracic duct. Batson's venous plexus extends from the skull to the sacrum. This valveless system theoretically offers less resistance to the spread of tumour emboli, especially when there is an increase in intrathoracic and intra-abdominal pressure, allowing retrograde flow by-passing pulmonary filters [5].

Nephrectomy may be justified in patients with metastatic disease to improve quality of life or local symptoms and to confer a possible survival advantage [6]. However, it is not justified when the intention is to induce spontaneous tumour regression which occurs in less than 1% of cases.

Management of tongue metastasis is surgical excision and this was followed in our patient by adjuvant radiotherapy to achieve local control of disease. Chemotherapeutic agents including fluoropyrimidines together with biological agents such as interferon-α can offer a palliative benefit in some patients with RCC. Shibayama *et al. *reported a complete response in a base of the tongue metastasis after interferon-α therapy [7]. Newer agents such as sorafenib and sunitinib have been shown to improve progression-free survival in metastatic RCC [8,9]. Temsirolimus and bevacizumab have also shown promise in early phase trials.

A thorough evaluation to distinguish between primary and secondary tongue cancer is essential. Primary cancer of the tongue is treated with curative intent and this includes total glossectomy with or without neck node dissection followed by radical radiotherapy in the early stages and concomitant chemotherapy (cisplatinum and 5 fluorouracil) and radiotherapy in the later stages.

Secondary tumours of the tongue are managed with palliative intent, which includes surgery, radiotherapy and immunotherapy. The prognosis of metastatic RCC is poor and 5-year survival is less than 10%.

## Conclusion

Twenty-eight case reports of tongue metastasis from kidney cancer since 1911 have been documented (Table 1) and its occurrence as a presentation of kidney cancer was found to be extremely rare with only three cases reported in the last century (Table 2) before the current case.

Metastatic spread to the tongue may occur in advanced stages of RCC. The prognosis of patients with tongue metastasis is poor because most of them have widespread disease. Similar to primary tumours of the tongue, metastatic lesions may be ulcerated or polypoid. Clinical and even histological differentiation between the two conditions can be challenging. Since the tongue is a rare metastatic site, when a lesion is detected, a thorough evaluation should be made to distinguish between metastasis and primary cancer, so that appropriate treatment can be offered.

## Abbreviations

RCC: Renal cell carcinoma; MRI: Magnetic resonance imaging; CT: Computed tomography; MDT: Multidisciplinary team.

## Competing interests

The authors declare that they have no competing interests.

## Authors' contributions

FA assisted in the conception and design of the paper, and also helped in the acquisition, review and interpretation of the data. MA contributed towards data collection and drafting of the manuscript. SG was involved in conception, reviewing and finally approving the version to be published. All authors read and approved the final manuscript.

## Consent

Written and informed consent was obtained from the patient for publication of this case report and any accompanying images. A copy of the written consent is available for review by the Editor-in-Chief of this journal.
